# Assessment of Parental Risk Factors Involved in Orofacial Clefts in Hail, Saudi Arabia: A Retrospective Cohort Study

**DOI:** 10.7759/cureus.66962

**Published:** 2024-08-15

**Authors:** Talal A Alshammary, Majed M Alshammari, Yosef A Alanazi, Ruqayyah H Almarshdi, Atheer K K Alshammri, Lamia H Alharbi, Tariq M Alenezi, Eyad A Almaghuthwi

**Affiliations:** 1 Oral and Maxillofacial Department, King Khaled Hospital - Hail, Hail, SAU; 2 Oral and Maxillofacial Department, University of Hail College of Dentistry, Hail, SAU; 3 Medicine Department, University of Hail College of Dentistry, Hail, SAU

**Keywords:** environmental factor, saudi arabia, parental risk factors, congenital anomalies, orofacial clefts

## Abstract

Background

Orofacial clefts are congenital anomalies affecting the development of the oral and facial structures, influenced by genetic and environmental factors. The prevalence of orofacial clefts varies globally, necessitating region-specific studies to understand contributing factors. Orofacial clefts are among the most common congenital defects affecting the head and neck, underscoring the importance of investigating paternal and maternal influences on their development to enhance awareness and understanding of potential contributing factors. Therefore, this research aimed to investigate parental risk factors contributing to the development of orofacial clefts.

Methods

A retrospective cohort study was conducted at the Oral and Maxillofacial Department of King Khaled Hospital, Hail, Saudi Arabia, involving 40 parents of children born between 2019 and 2023 with orofacial clefts. Data collection included interviewer-administered questionnaires with parents addressing demographic information, pregnancy details, parental medical history, and postoperative outcomes. Statistical analysis utilized descriptive statistics, chi-square tests, Fisher's exact test, and linear regression, with significance defined as p<0.05.

Results

The study had a gender distribution of 19 males (47.5%) and 21 females (52.5%) among orofacial cleft cases (p<0.75), with cleft palate (13 cases, 32.5%) and cleft lip (11 cases, 27.5%) being the most prevalent anomalies (p<0.05). Maternal supplementation rates were high, with 34 mothers (85%, p<0.05) taking folic acid and 36 mothers (90%, p<0.05) taking iron, yet orofacial clefts incidence persisted. Paternal risk factors such as tobacco use were reported by 19 fathers (47.5%, p<0.05), and familial history of orofacial clefts was noted in nine cases (22.5%, p<0.05). Postoperative outcomes indicated varying levels of functional recovery and satisfaction.

Conclusion

This study explored the complex origins of orofacial clefts, emphasizing genetic and environmental influences. The findings suggest a potential paternal risk factor. The study highlights the need for further investigation into genetic mechanisms and the development of effective prevention strategies.

## Introduction

The development of the orofacial region relies on intricate tissue processes that must synchronize precisely to form the oral cavity and facial structures, a complex natural process. Orofacial clefts can result from disruptions in the fusion or development of facial processes, particularly the maxillary and medial nasal processes [1]. The areas most commonly affected by clefts include the alveolar ridge, palate (both hard and soft), and upper lip, with less frequent involvement of the eyes and nose [2]. Clefts are categorized into syndromic, affecting multiple developmental areas, and non-syndromic, affecting a single area [3]. Data from various countries indicate an increasing prevalence of orofacial cleft cases at birth, rising from 1 per 1000 live births in the early 20th century to approximately 1.5 to 2 per 1000 live births in recent years [4]. Orofacial clefts, such as cleft lip, cleft palate, and cleft lip with cleft palate, encompass a diverse range of abnormalities that vary in causes, severity, and health impacts [5].

In the Hail region, the overall prevalence rate of orofacial clefts was found to be 1.08 per 1000 births. Among 30 diagnosed patients, 14 (46.7%) had cleft palate, 11 (36.7%) had bilateral cleft lip and palate, 4 (13.3%) had bilateral cleft lip, and only one (3.3%) had unilateral cleft lip and palate [6]. The occurrence of orofacial clefts is believed to be multifactorial, influenced predominantly by genetic and environmental factors [7]. Identifying factors associated with orofacial clefts is crucial for developing preventive strategies, including genetic counseling and public education on nutritional factors, tobacco use, and alcohol consumption [8]. Orofacial clefts are among the most common congenital defects affecting the head and neck, underscoring the importance of investigating paternal and maternal influences on their development to enhance awareness and understanding of potential contributing factors. Comprehensive understanding and investigation of this congenital defect are essential due to its potential to complicate patients' lives. Therefore, this research aimed to investigate parental risk factors contributing to the development of orofacial clefts. 

## Materials and methods

A retrospective cohort study was conducted with 55 participants at the Oral and Maxillofacial Department of King Khaled Hospital in Hail, Saudi Arabia. The aim of this study was to investigate parental risk factors contributing to the development of orofacial clefts. We achieved this through the following objectives: identifying and categorizing parental risk factors, assessing environmental exposures, and identifying the most commonly presented types of clefts. The research involved interviewer-administered questionnaires targeting parents of children diagnosed with orofacial clefts in a hospital setting from 2019 to 2023. The questionnaire addressed various factors, including sex, age, type of orofacial cleft, medication use during pregnancy, pregnancy complications, physical and mental health during pregnancy, parents’ medical history (systemic diseases, family history of orofacial clefts, history of miscarriage, tobacco consumption or exposure, and parental consanguinity), and postoperative outcomes of patients.

According to a review of the patient files, 55 individuals visited the department for reasons related to orofacial clefts; 15 of them were excluded due to being born before 2019. Phone interviews were conducted with 40 parents to complete the questionnaire after obtaining informed consent. To ensure validity, each co-author collecting information wrote their and the patient’s names in the designated box on the questionnaire. A month later, the principal investigator randomly selected two questionnaires from each co-author, re-contacted those participants, and obtained the information again. The data were then compared to the initial responses, which showed matching results. Subsequently, data were collected, recorded, and coded for statistical analysis. Inclusion criteria comprised children diagnosed with orofacial clefts born between 2019 and 2023 who voluntarily agreed to participate. Exclusion criteria included any discrepancy with the aforementioned inclusion criteria.

Statistical analysis

Descriptive statistics were utilized to elucidate qualitative variables, including demographic characteristics (gender and age). While investigating research inquiries, the chi-square test and linear regression were used through IBM SPSS Statistics for Windows, Version 21, (Released 2012; IBM Corp., Armonk, New York, United States). Statistical significance was defined by a p<0.05.

Ethical considerations

Ethical approval was obtained after submitting a fully detailed proposal to the Research and Study Department and the Institutional Review Board at Hail Health Cluster with approval log number 2024-16. Informed consent was obtained from all participants, ensuring their understanding of the purpose and voluntary participation in both hospital data collection and the interview process. 

## Results

There were 55 participants initially selected for the present study; however, 15 children were excluded as they were born before 2019. In the end, 40 children were chosen to contact their parents for the present study. The study investigated the characteristics of the participant population, revealing a gender distribution of 47.5% males and 52.5% females, with a non-significant difference (p=0.75). 

**Figure 1:**
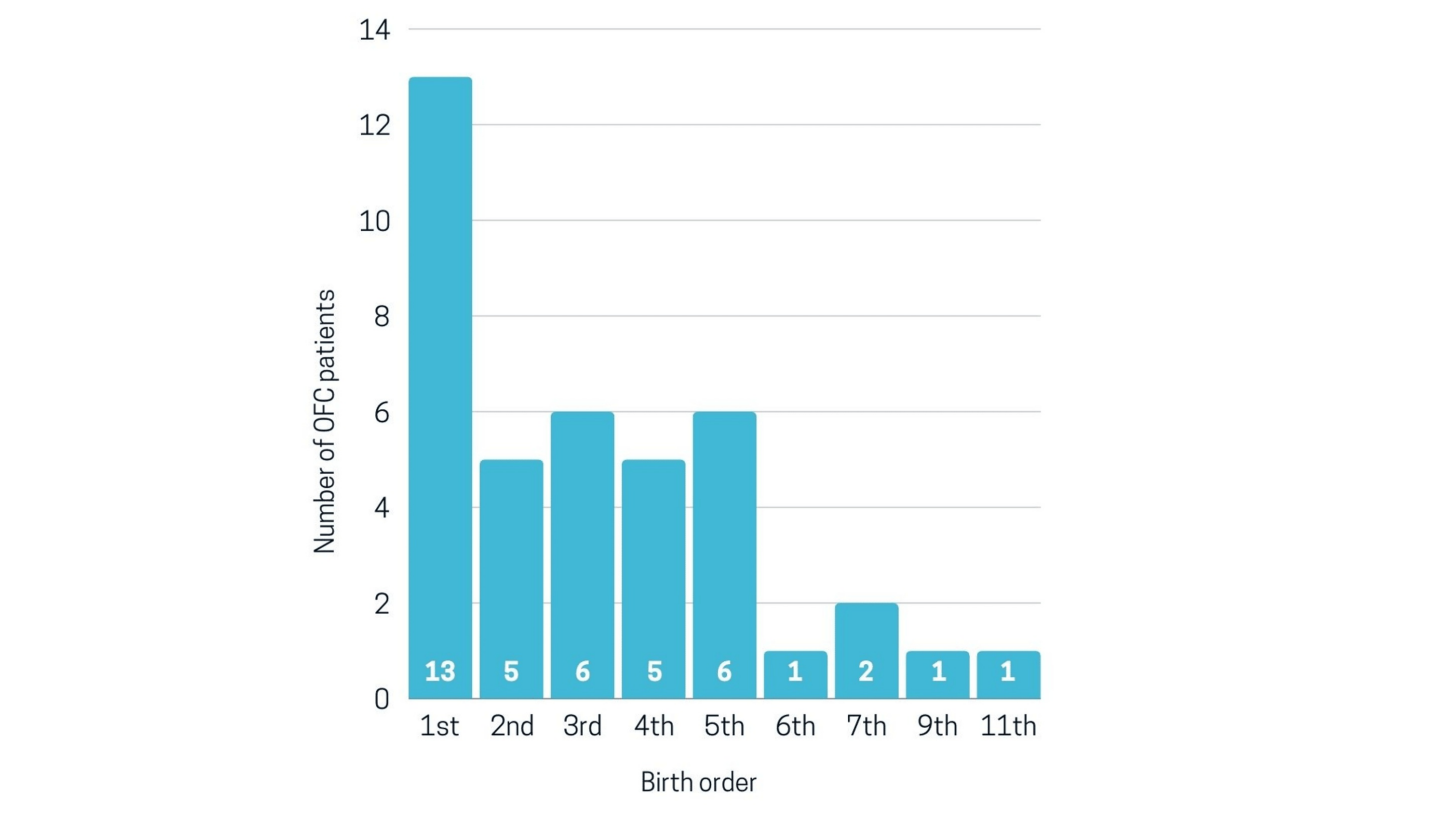
Birth order distribution

The age distribution demonstrated that the mean age of the participants was 2.69±1.11 years, and most of the participants were two to four years old (60%), followed by <two years (32.5%), and 7.5% of the participants were four to six years old with a significant difference among the age groups (p<0.05). Most of the children were firstborns (32.5%), followed by the fifth borns (15%), while the least in number were sixth, ninth, and eleventh borns (2.5% for each birth order), and a significant difference was observed (p<0.05) (Figure 1). 

**Figure 2:**
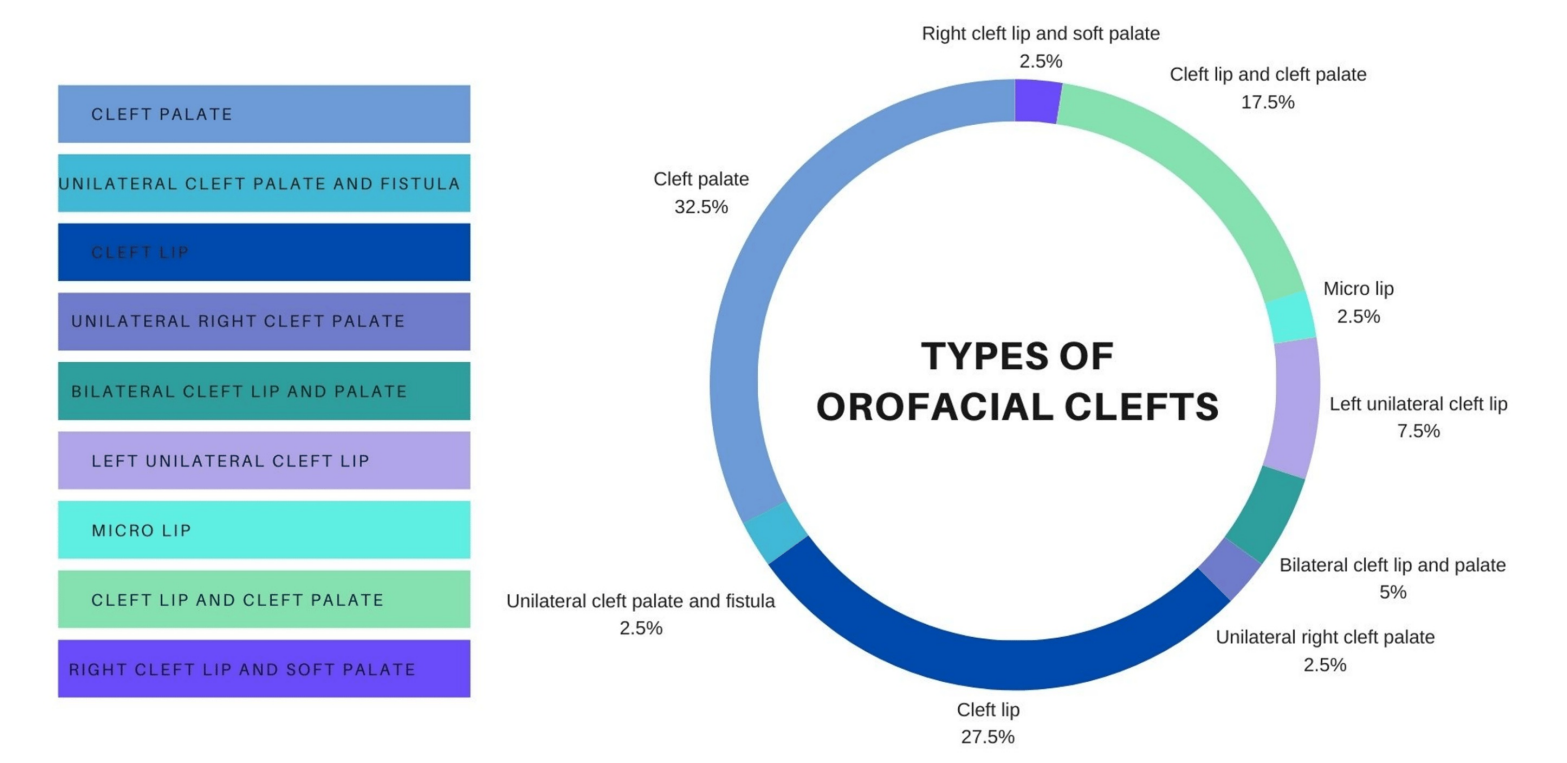
Distribution according to orofacial cleft (OFC) types

In terms of types of orofacial cleft, most of the children had cleft palate (32.5%), followed by cleft lip (27.5%), and cleft lip and cleft palate (17.5%). The least number of children were with bilateral cleft lip and palate, unilateral cleft palate and fistula, right cleft lip and soft palate, and unilateral cleft palate right (2.5% for each type), followed by micro lip (5%), and left unilateral cleft lip (7.5%) with a significant difference (p<0.05) as determined by a chi-square test for independence (Figure 2). 

There were different types of medications utilized by pregnant women during pregnancy, and most of the women took iron (90%, p<0.05), followed by folic acid (85%, p<0.05), vitamins (75%, p<0.05), and 40% of women took other medications such as calcium, heparin, etc. (p=0.33); 15% of the women (p<0.05) took antibiotics during pregnancy. Furthermore, a very few number and non-significant (p=0.98) number of women were reported with complications, and 5% each were reported with bleeding and fever (Table 1).

**Table 1:** Age of children and health/supplement intake of mothers during pregnancy *statistically significant

Characteristic	N (%)	p-value
Age (years)		
<2	13 (32.5%)	0.032*
2-4	24 (60%)	
4-6	3 (7.5%)	
Medication during pregnancy		
Folic acid		
Yes	34 (85%)	0.041*
No	6 (15%)	
Antibiotics		
Yes	6 (15%)	0.027*
No	34 (85%)	
Iron		
Yes	36 (90%)	0.018*
No	4 (10%)	
Vitamins		
Yes	30 (75%)	0.035*
No	10 (25%)	
Others		
Yes	16 (40%)	0.044*
No	24 (60%)	
Complications during pregnancy		
Bleeding	2 (5%)	0.98
Fever	2 (5%)	
Placenta abruption	1 (2.5%)	
Corona	1 (2.5%)	
Chest allergy	1 (2.5%)	
Anaemia	1 (2.5%)	
Regurgitation	1 (2.5%)	

Table 2 presents the physical and mental health conditions observed in women during pregnancy. Depression was the most frequently observed condition, affecting 30% of women. Anxiety and fatigue were each reported in 27.5% of women. Hyper/hypotension and hyper/hypoglycemia were found in 15% and 10% of cases, respectively. The differences across these conditions were not statistically significant (p>0.05) (Table 2).

**Table 2:** Physical and mental health during pregnancy

Characteristic	N and percentage	p-value
Depression	12 (30%)	0.15
Anxiety	11 (27.5%)	0.31
Fatigue	11 (27.5%)	0.31
Hypertension/hypotension	6 (15%)	0.64
Hyperglycemia/hypoglycemia	4 (10%)	.073

Table 3 summarizes the family medical history from both the mother’s and father’s perspectives. For mothers, there were no significant differences observed (p>0.05) across various parameters: 30% had a history of miscarriages, 17.5% had a family history of orofacial cleft, and 5% had systemic diseases such as cardiovascular disease and diabetes. Additionally, 12.5% of mothers were exposed to tobacco smoke externally. From the father’s perspective, a significant difference was noted (p<0.05) for tobacco consumption, with 47.5% of fathers reporting this habit. No significant differences were observed for endogamy (32.5%), family history of orofacial cleft (22.5%), or systemic diseases (10%) (Table 3).

**Table 3:** Family medical history *statistically significant

Characteristic	N and percentage	p-value
Mother's medical history		
Systemic diseases	2 (5%)	0.33
Family history of orofacial	7 (17.5%)	0.94
Miscarriage’s history	12 (30%)	0.73
External tobacco smoking exposure	5 (12.5%)	0.98
Father's medical history		
Endogamy	13 (32.5%)	0.53
Systemic diseases	4 (10%)	0.73
Family history of orofacial	9 (22.5%)	0.94
Tobacco consumption	19 (47.5%)	0.001*

After the successful surgery, overall, a significant difference (p<0.05) was observed in the performance and satisfaction with the postoperative outcomes, with 75% of the children being able to eat and drink, while 25% did not report any outcomes. Similarly, 55% of the children were able to speak, and 70% of the parents were satisfied with the outcomes, while only 5% were not satisfied (Table 4).

**Table 4:** Postoperative outcomes *statistically significant

Characteristic	N (%)	p-value
Child able to eat and drink		
Yes	30 (75%)	0.03*
Not reported	10 (250%)	
Child able to speak		
Yes	22 (55%)	0.02*
No	8 (20%)	
Not Available	10 (25%)	
Overall satisfaction		
Yes	28 (70%)	0.01*
No	2 (5%)	
Not Available	10 (25%)	

Different factors such as gender, age, birth order, physical and mental health during pregnancy, and family (mother's and father's) history of orofacial cleft associated with the occurrence of orofacial cleft in child outcomes are presented as regression coefficients with 95% confidence interval (CI) in Table 5. Gender had a non-significant positive association with orofacial cleft (β=0.226, p=0.26) while, age and birth order had a non-significant negative association (β=-0.024, p=0.89 and β=-0.824, p=0.41, respectively). In terms of physical and mental health, hyper/hypotension had a significant negative association (β=-2.812, p=0.01) with orofacial cleft in children. Meanwhile, the remaining variables had a non-significant association with orofacial cleft (Table 5). In the family medical history from the mother’s perspective, there was a non-significant positive (external tobacco smoking exposure) and negative (systemic diseases, family history of orofacial cleft, and miscarriage) association. From the father’s medical history perspective, a significant negative association (β=-0.421, p=0.03) was observed in the family history of orofacial cleft with orofacial cleft in children, while a non-significant association was observed among the remaining variables (Table 5).

**Table 5:** Linear regression to explore different variables with the types of orofacial cleft

Variable	Standardized coefficient	t-value	Significance	95.0% confidence interval (CI)	
	Beta			Lower bound	Upper bound
Gender	0.226	1.15	0.26	-0.80	2.83
Age	-0.024	-0.138	0.89	-0.801	0.70
Birth order	-0.136	-0.824	0.418	-0.496	0.213
Physical/mental health during pregnancy					
Fatigue	0.320	1.762	0.091	-0.280	3.498
Hypertension/hypotension	-0.506	-2.812	0.010	-5.522	-0.841
Hyperglycemia/hypoglycemia	-0.173	-0.774	0.447	-4.757	2.167
Depression	0.159	0.543	0.592	-2.189	3.747
Anxiety	0.133	0.616	0.544	-1.582	2.925
Mother's medical history					
Systemic diseases	-0.155	-0.663	0.514	-6.568	3.380
Family history of orofacial	-0.079	-0.376	0.710	-3.028	2.097
History of miscarriages	-0.162	-0.921	0.367	-2.583	0.992
External tobacco smoking exposure	0.145	0.81	0.423	-1.509	3.47
Father's medical history					
Endogamy	-0.209	-1.150	0.262	-2.803	0.800
Systemic diseases	0.121	0.614	0.545	-2.140	3.94
Family history of orofacial cleft	-0.421	-2.189	0.039	-4.480	-0.125
Tobacco consumption	0.064	0.323	0.750	-1.1558	2.134

## Discussion

The aim of this study was to investigate the paternal and maternal influences on the development of orofacial clefts through structured interviews with the parents. Orofacial clefts are defects that can arise due to various factors, particularly genetics and environmental risk factors [9].

Among 40 participants, comprising 47.5% males and 52.5% females, cleft palate was the most commonly found anomaly (32.5%), consistent with research conducted in 2017 by AlShammari et al., who reported cleft palate as the most common type among orofacial clefts in northern Saudi Arabia [6]. Following cleft palate were cleft lip (27.5%), cleft lip and palate (17.5%), with bilateral cleft lip and palate, unilateral cleft palate and fistula, right cleft lip and soft palate, and right unilateral cleft palate being the least common (2.5% each), followed by micro lip (5%) and left unilateral cleft lip (7.5%).

Numerous studies have indicated that supplementation of women with folic acid during early pregnancy reduces the likelihood of experiencing unfavorable outcomes such as birth abnormalities [10]. However, our findings contradict this point, as 85% of orofacial cleft patients' mothers had folic acid supplementation during pregnancy, yet orofacial clefts still occurred (p<0.05).

Hozyasz et al. mentioned in their study that the likelihood of orofacial clefts in offspring is reduced with multivitamin intake around the time of conception, although caution is advised against excessive consumption of preformed vitamin A (retinol) [11]. In contrast, our findings showed that 75% of mothers took vitamin supplements during pregnancy, suggesting that vitamin deficiencies may not increase the risk of orofacial cleft occurrence (p<0.05).

Complications such as bleeding, fever, placenta abruption, COVID-19, chest allergy, anemia, and regurgitation showed no significant correlation with the development of orofacial clefts (each with a prevalence of 2.5% to 5%, p=0.98). In contrast, the study by Cheshmi et al. (2020) identified significant associations between orofacial clefts and broader maternal complications such as preeclampsia, anemia, pregnancy hypertension, gestational diabetes, and intrauterine hypoxia, all of which were found to significantly increase the risk of orofacial clefts [12].

Our study identified an insignificant association between maternal depression (30%) and the incidence of orofacial clefts in newborns. Additionally, our study did not find significant associations for anxiety (27.5%) and fatigue (27.5%), which contradicts the findings in Sharma et al.'s study, which showed that maternal mental health issues, such as depression, anxiety, and fatigue, significantly impacted fetal development, including the development of orofacial clefts [13].

Genetics plays a significant role in the development of orofacial clefts, as demonstrated by Babai and Irving in their study on orofacial clefts. Cases of orofacial clefts were reported in 22.5% of the children with a paternal history and 17.5% with a maternal history of orofacial clefts. Additionally, there was a notable correlation between orofacial cleft occurrence and cases of endogamous marriages, accounting for 30% of orofacial cleft cases. Therefore, further genetic analysis is necessary to elucidate the role of genetics in orofacial clefts [14].

A meta-analysis reported statistically significant and moderate correlations between maternal smoking and orofacial clefts [15]. However, only 12.5% of mothers reported external tobacco smoking exposure, which was statistically insignificant. Other studies have found no connection between maternal external tobacco smoking and orofacial cleft development [16].

Our study's results indicated no strong relationship between iron supplementation during pregnancy and reduced incidence of orofacial clefts, as 90% of orofacial cleft patients' mothers had iron supplementation. Conversely, evidence suggests that increased consumption of vegetable protein, fiber, iron, ascorbic acid, and magnesium may reduce the risk of orofacial clefts [17].

This study revealed that 32.5% of orofacial cleft patients were firstborns in their families, suggesting a relatively high chance of the first child having orofacial clefts compared to subsequent births. In contrast to the well-investigated maternal risk factors associated with orofacial clefts, paternal risk factors remain less explored. Our study suggests a notable correlation between paternal smoking, paternal history of orofacial clefts, and the occurrence of orofacial clefts. Specifically, 47.5% of fathers admitted to being habitual smokers, while no maternal tobacco consumption was reported. Moreover, cases of orofacial clefts were reported in 22.5% with a paternal history compared to 17.5% with a maternal history. These results may indicate a stronger association between paternal risk factors and orofacial clefts than maternal factors, suggesting a need for further investigation into paternal risk factors associated with orofacial clefts.

Limitations

This study had several limitations, including a small sample size, retrospective design in the absence of a control group, and potential recall bias in parental interviews. Future research with larger, multi-center cohorts and prospective data collection is necessary to validate our findings and explore additional risk factors.

## Conclusions

This study elucidates the multifaceted origins of orofacial clefts, underscoring the interplay of genetic and environmental factors. Among 40 participants, with no significant difference in terms of gender, cleft palate was the predominant anomaly. The findings suggest a potential paternal risk factor associated with orofacial clefts. Despite mothers receiving their supplements during pregnancy, these measures did not appear to significantly decrease the incidence of orofacial clefts. Further investigation is crucial to elucidate the genetic mechanisms involved and to develop effective prevention strategies.
